# Supplementary material for: RNA-DNA strand exchange by the Drosophila Polycomb complex PRC2
Source: Nat Commun. 2020 Apr 14;11:1781. doi: 10.1038/s41467-020-15609-x (PMC7156742; doi:10.1038/s41467-020-15609-x)
Supplement: Supplementary file 1 — Supplementary Information [file 41467_2020_15609_MOESM1_ESM.pdf]

RNA-DNA strand exchange by the *Drosophila* Polycomb complex PRC2

Alecki *et al.*

Supplementary information

Supplementary contents:

Supplementary figures 1-12

Supplementary tables 1-3

a

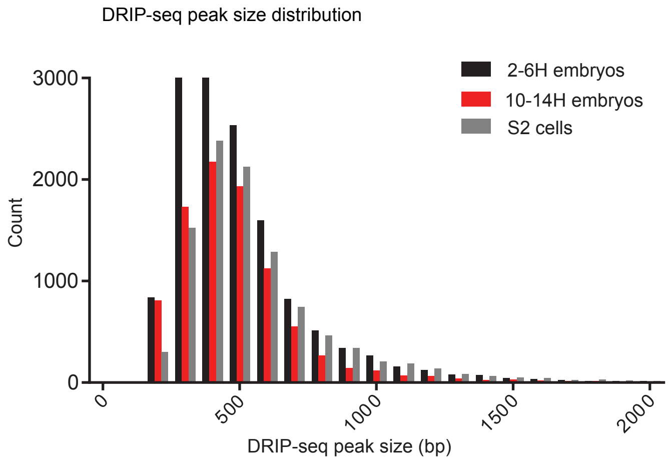

b

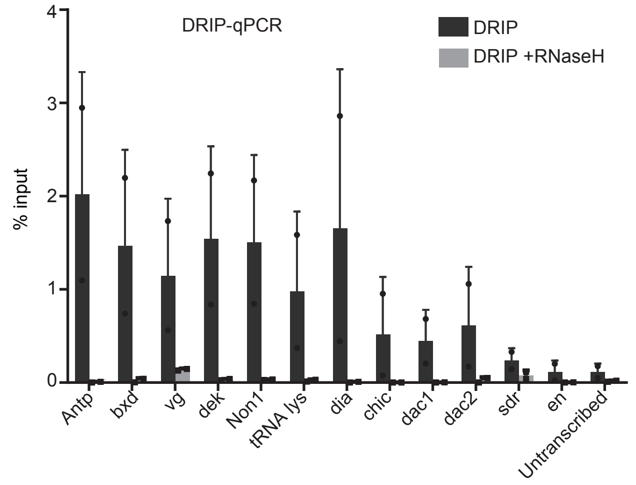

c

	R-loop peaks	Overlap with gene body	Overlap with EST
2-6H embryos	11233	8479 (75%)	9090 (81%)
10-14H embryos	7471	5378 (72%)	5675 (76%)
S2 cells	9605	7258 (76%)	7380 (77%)

d

	PREs overlapping R-loops	p-value (permutation analysis)
2-6H embryos	180/541 (33%)	0.001
10-14H embryos	118/541 (22%)	0.001
S2 cells	156/541 (29%)	0.001

e

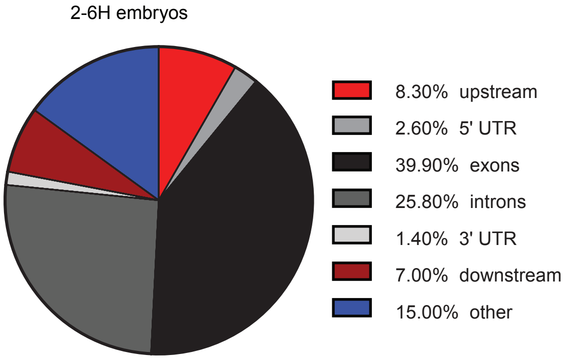

f

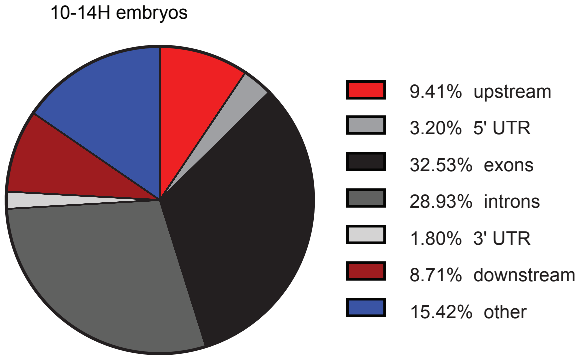

g

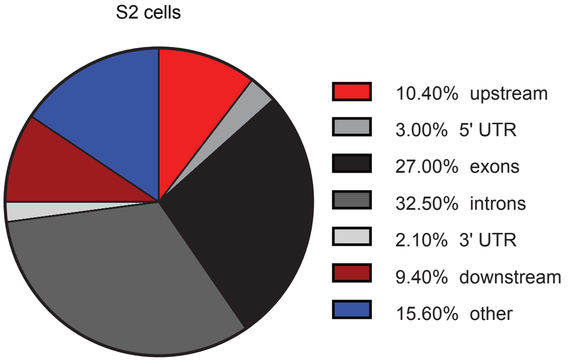

h

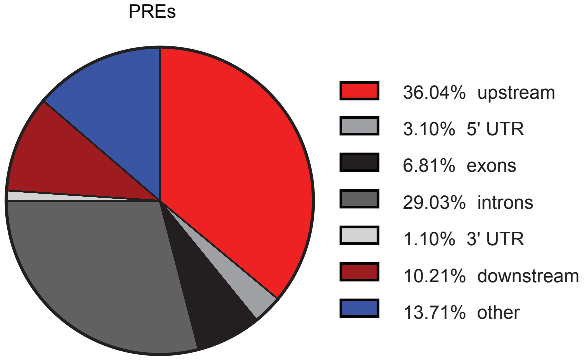

Supplementary Fig. 1 Overview of DRIP-seq analysis. a. Distribution of DRIP-seq peak sizes. b. 10 positive sites and 3 negative sites were confirmed by DRP-qPCR using S2 cell genomic DNA with or without RNaseH treatment. Graph show the mean +/- S.D. c. Summary of overlaps of R-loops with genes. Note that heterochromatin, ChrU, and mitochondrial genome sequences were removed for this analysis. d. Summary of overlaps of R-loops with PREs. e-h. Pie charts of distribution of R-loops (e-g) and PREs (h) across the genome. Source data are provided in Source Data file.

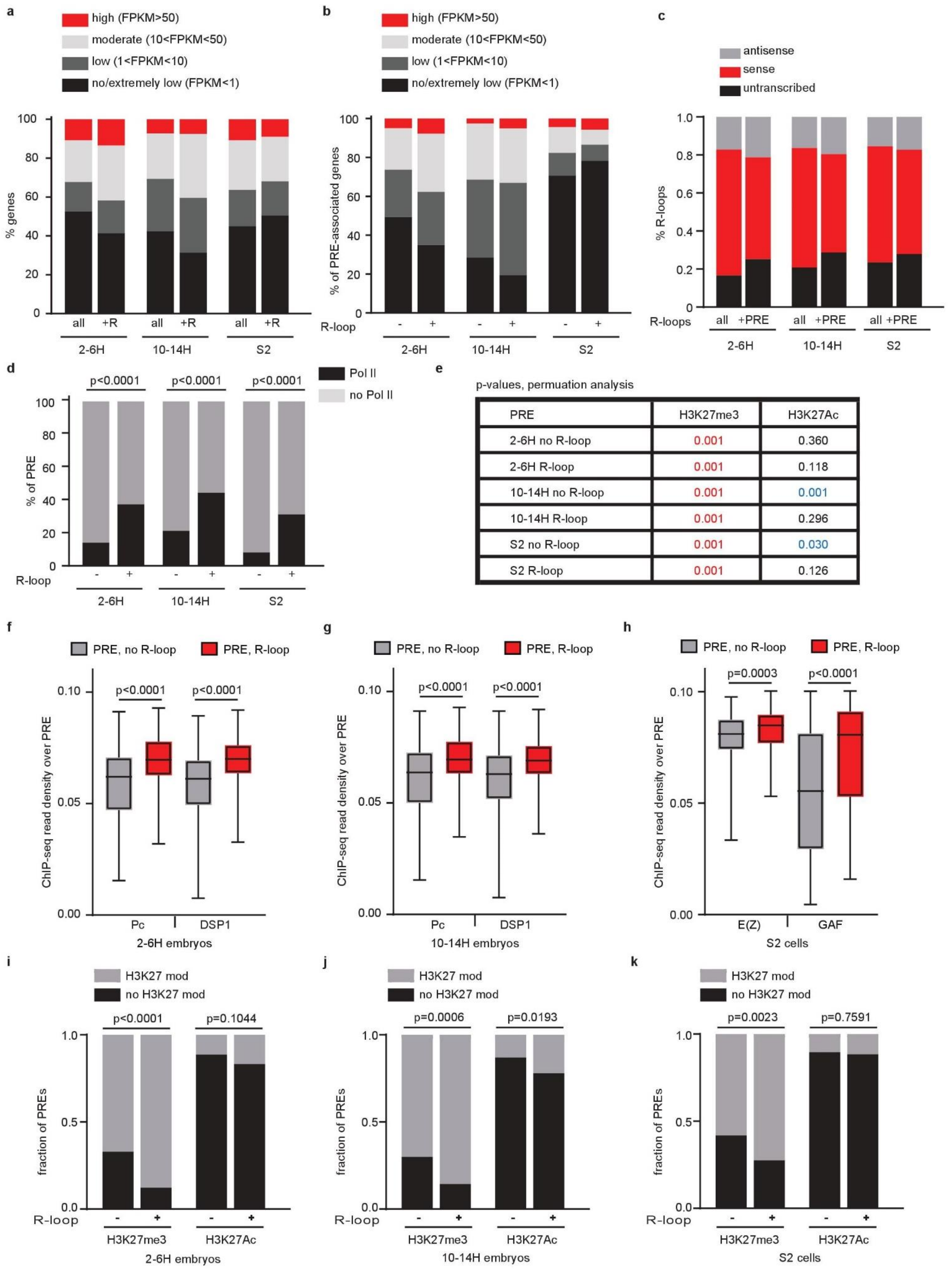

p-values, permuation analysis

PRE	H3K27me3	H3K27Ac
2-6H no R-loop	0.001	0.360
2-6H R-loop	0.001	0.118
10-14H no R-loop	0.001	0.001
10-14H R-loop	0.001	0.296
S2 no R-loop	0.001	0.030
S2 R-loop	0.001	0.126

Supplementary Fig. 2 Relationship between R-loop formation, transcription, and PcG proteins. a. Expression levels of all genes as compared with genes with R-loops. b. Expression level of genes associated with PREs that do not or do form R-loops. c. Strandedness of all R-loops or R-loops formed at PREs relative to annotated transcripts. R-loops at PREs are more likely to only overlap antisense transcripts in 2-6H (p=0.002) and 10-14H (p=0.0117) data sets by Fisher's exact test. Note that "antisense" means that transcription is only annotated in the antisense orientation, but many R-loops that overlap a "sense" transcript that could explain the R-loop also have annotated antisense transcripts. d. Fraction of PREs that do or do not form R-loops that overlap RNA polymerase II peaks. p-values are for Fisher's exact test comparing the overlap for PREs that do or do not form R-loops. e. Summary of overlaps between PREs that do or do not form R-loops and H3K27me3 or H3K27Ac ChIP-seq peaks. Red numbers indicate that overlap is higher than random and blue numbers that it is lower than random. Overlap between PREs with or without R-loops is not significant, and is in fact anticorrelated in some cases (blue numbers). f-h. Median density of normalized ChIP-seq reads over PREs that do or do not form R-loops. Whiskers show min. to max. i-k. Fraction of PREs that do or do not form R-loops that overlap H3K27me3 or H3K27Ac peaks. p-values are for Fishers exact test comparing the overlap for PREs that do or do not have R-loops.

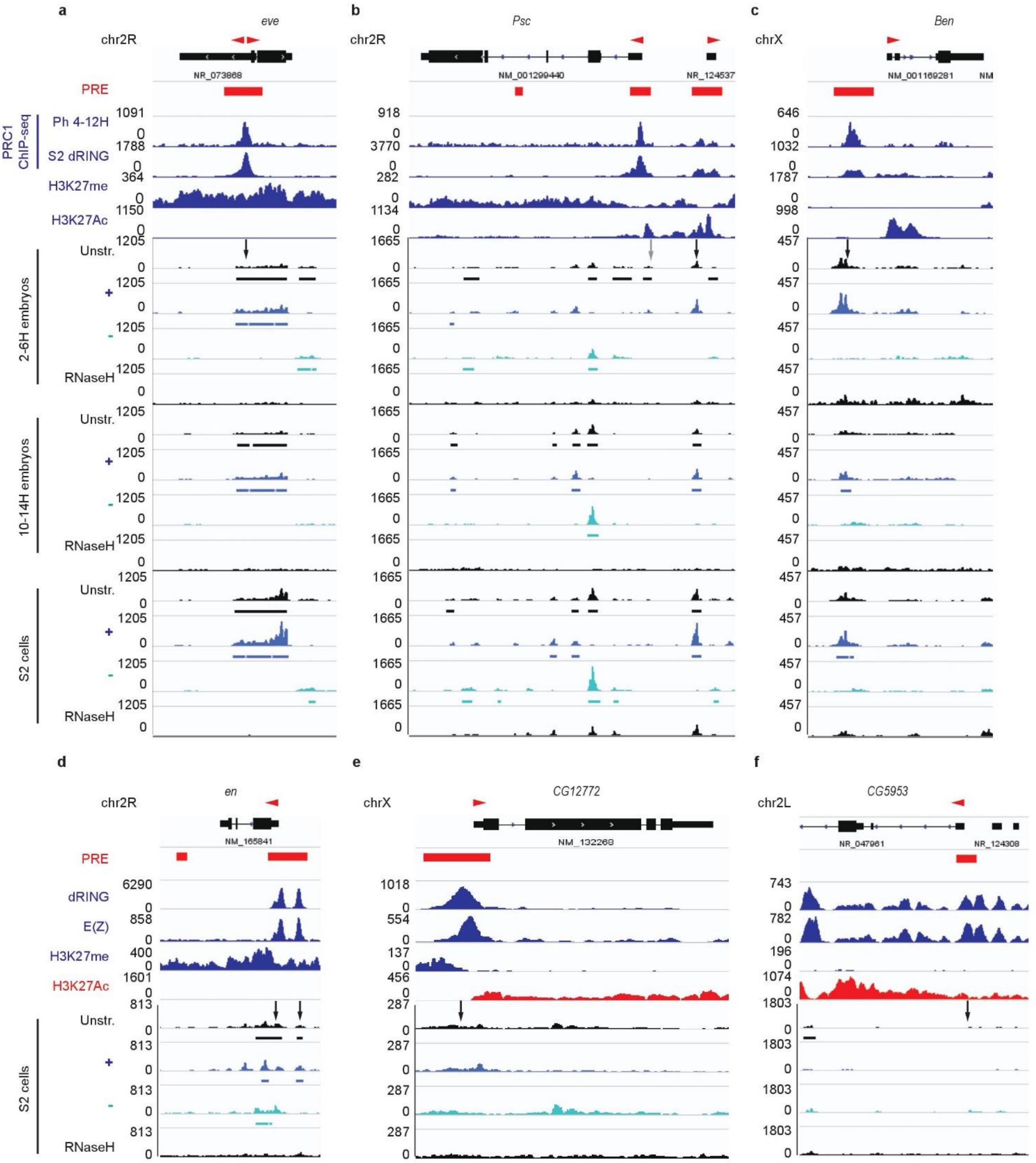
a
eve
chr2R
NR_073868
PRE
PRC1 ChIP-seq
Ph 4-12H
S2 dRING
H3K27me
H3K27Ac
2-6H embryos
10-14H embryos
S2 cells
Unstr.
+
-
RNaseH
b
Psc
NM_001299440
NR_124537
c
Ben
chrX
NM_001169281
d
en
NM_165841
dRING
E(Z)
e
CG12772
NM_132269
f
CG5953
chr2L
NR_047961
NR_124308

Supplementary Fig. 3 Additional examples of R-loops. a. An R-loop forms over the PRC1-bound PRE with the same strandedness as *eve* transcription (arrow), but opposite to the direction of the overlapping ncRNA. b. An R-loop forms at a PRC1-bound PRE at the *Psc* gene. Note that an R-loop is detected at the PRE overlapping the promoter in 2-6H embryos (grey arrow), while R-loop signal is detected in all samples at the upstream PRE that overlaps the non-coding RNA (black arrow). c. Arrow indicates R-loop that overlaps a PRC1-bound PRE upstream of the *ben* gene but has no annotated transcript. d-f. Relationship between H3K27 modification, R-loops, and PREs in S2 cells. d. The *en* PRE has characteristics of the OFF state: the PRE has high levels of H3K27me3, both PRC1 and PRC2 binding, and forms R-loops (arrows). e. The PRE at the promoter for *CG12772* has characteristics of a balanced state, with both H3K27Ac and H3K27me3 modifications, PRC1 and PRC2 binding, and no R-loop (arrow). f. The PRE at *CG5953* has characteristics of the ON state, with PRC1 binding, high levels of H3K27Ac, no H3K27me3, and no R-loop formation. RNA-seq data (FPKM) are consistent with these states: *en*=0.016, *CG12772*=6.3, *CG5953*=3.6, 15.6 (2 isoforms).

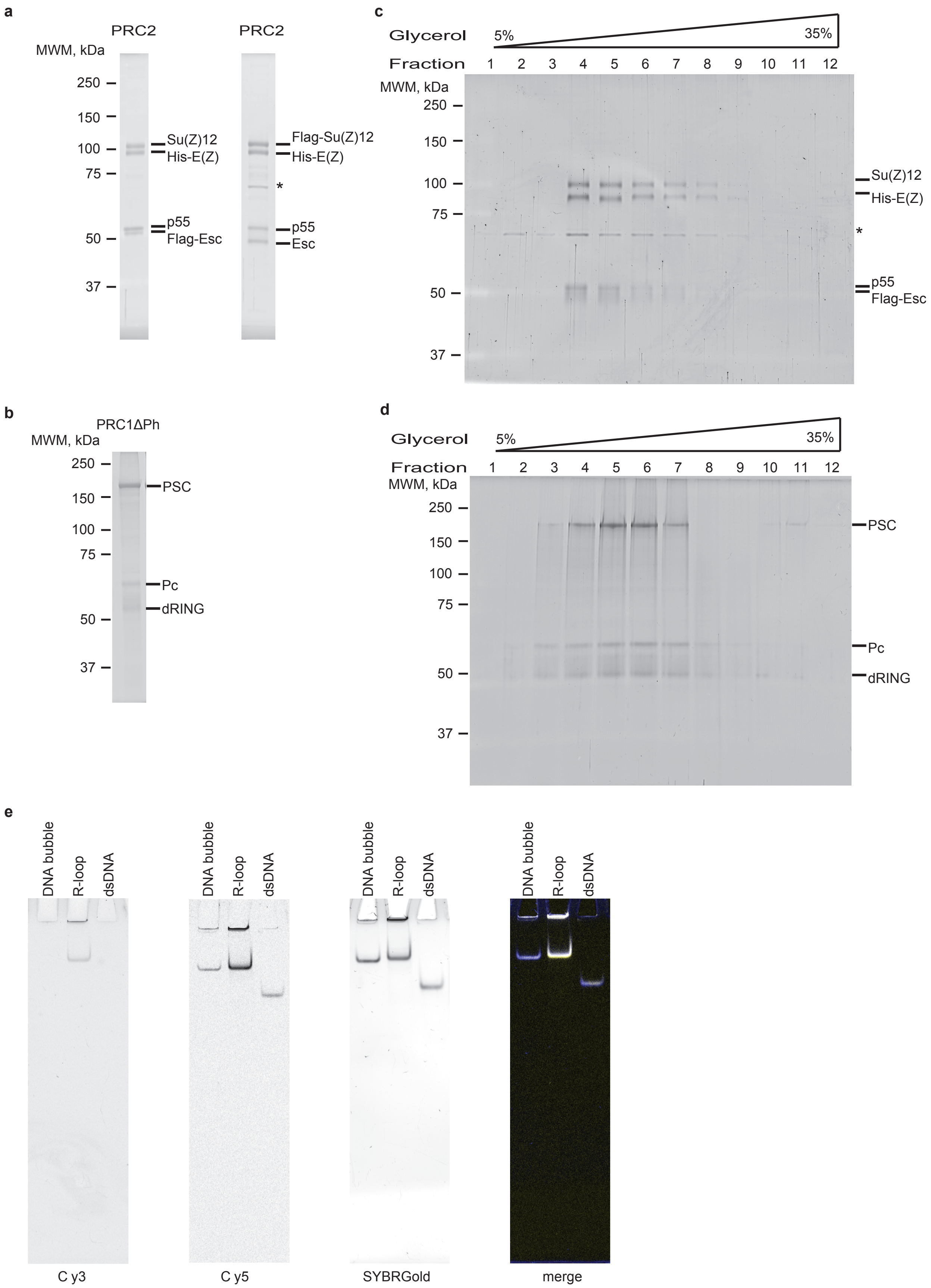
a
PRC2
PRC2
MWM, kDa
250
150
100
75
50
37
Su(Z)12
His-E(Z)
p55
Flag-Esc
Flag-Su(Z)12
His-E(Z)
*
p55
Esc
b
PRC1ΔPh
MWM, kDa
250
150
100
75
50
37
PSC
Pc
dRING
c
Glycerol
5%
35%
Fraction 1 2 3 4 5 6 7 8 9 10 11 12
MWM, kDa
250
150
100
75
50
37
Su(Z)12
His-E(Z)
*
p55
Flag-Esc
d
Glycerol
5%
35%
Fraction 1 2 3 4 5 6 7 8 9 10 11 12
MWM, kDa
250
150
100
75
50
37
PSC
Pc
dRING
e
DNA bubble
R-loop
dsDNA
C y3
C y5
SYBRGold
merge

Supplementary Fig. 4 Templates and proteins used to analyze PRC1 and PRC2 interactions with R-loops in vitro. a, b. SYPRO Ruby-stained SDS-PAGE of PRC2 (a) (10% acrylamide), and PRC1ΔPh (b) (8% acrylamide) used in this study. PRC2 prepared with Flag-Su(Z)12 or Flag-Esc were used interchangeably. *=HSC70 contaminant. c, d. SYPRO Ruby-stained SDS-PAGE of glycerol gradient fractions of PRC2 (c) or PRC1ΔPh (d). The predicted molecular weight of PRC2 is 284 kDa, and of PRC1ΔPh 261 kDa. e. Oligonucleotide substrates used for EMSA and filter binding. Note that these substrates were prepared with fluorescently labelled oligonucleotides and the unlabelled 5' ends subsequently labelled with ^{32}P for binding assays.

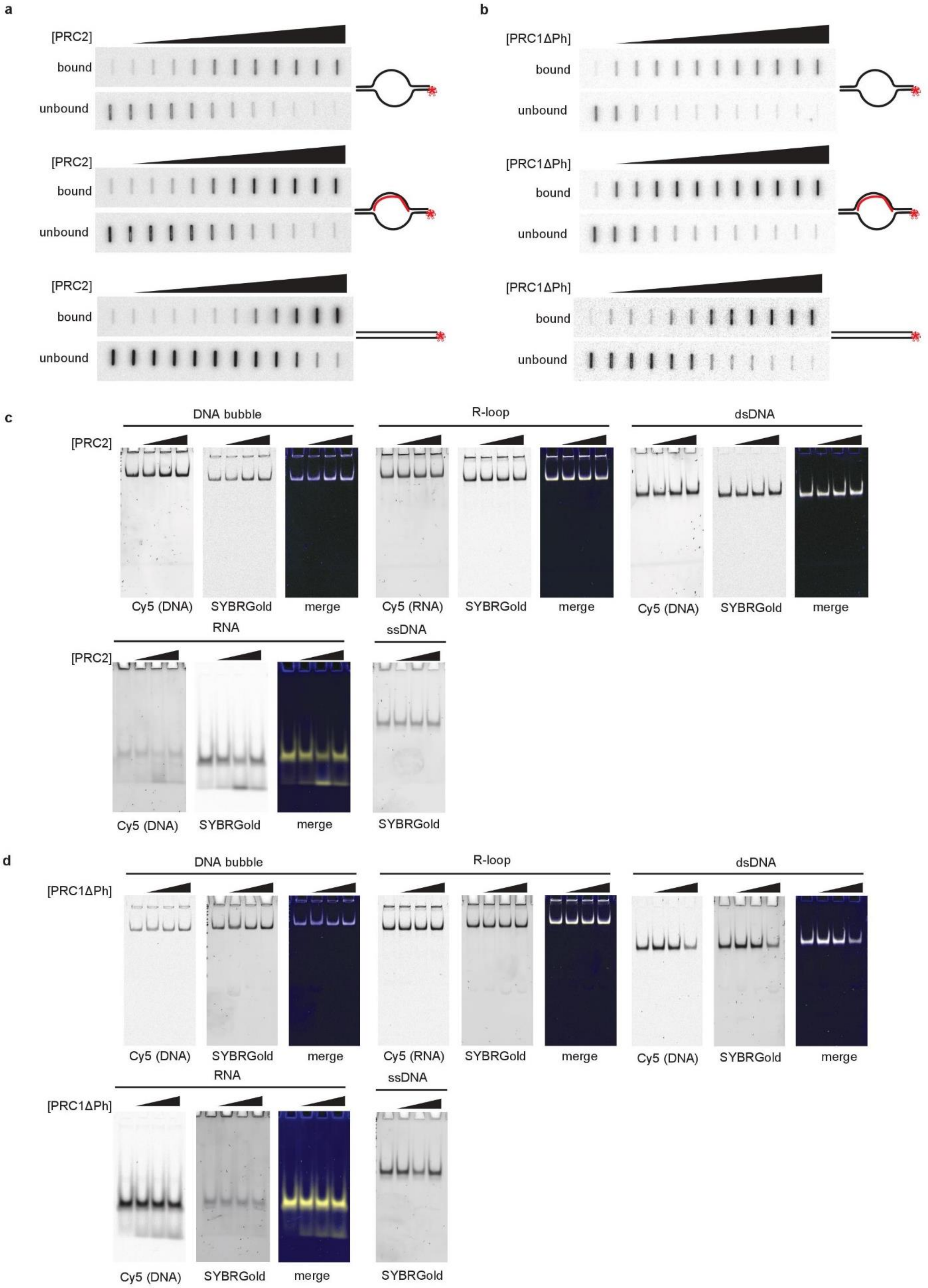
a
[PRC2]
bound
unbound
[PRC2]
bound
unbound
[PRC2]
bound
unbound
b
[PRC1ΔPh]
bound
unbound
[PRC1ΔPh]
bound
unbound
[PRC1ΔPh]
bound
unbound
c
DNA bubble
R-loop
dsDNA
[PRC2]
Cy5 (DNA)
SYBRGold
merge
Cy5 (RNA)
SYBRGold
merge
Cy5 (DNA)
SYBRGold
merge
RNA
ssDNA
[PRC2]
Cy5 (DNA)
SYBRGold
merge
SYBRGold
d
DNA bubble
R-loop
dsDNA
[PRC1ΔPh]
Cy5 (DNA)
SYBRGold
merge
Cy5 (RNA)
SYBRGold
merge
Cy5 (DNA)
SYBRGold
merge
RNA
ssDNA
[PRC1ΔPh]
Cy5 (DNA)
SYBRGold
merge
SYBRGold

Supplementary Fig. 5 PRC1 and PRC2 interactions with nucleic acids. a, b. Representative filter binding assay for the data presented in Fig. 2a, b. c, d. Native acrylamide gel analysis of oligonucleotide substrates after incubation with PRC2 (c) or PRC1ΔPh (d) under conditions used for filter binding assays, demonstrating that the substrates remain intact. PRC2 or PRC1ΔPh were incubated with 20 nM probe followed by Proteinase K digestion.

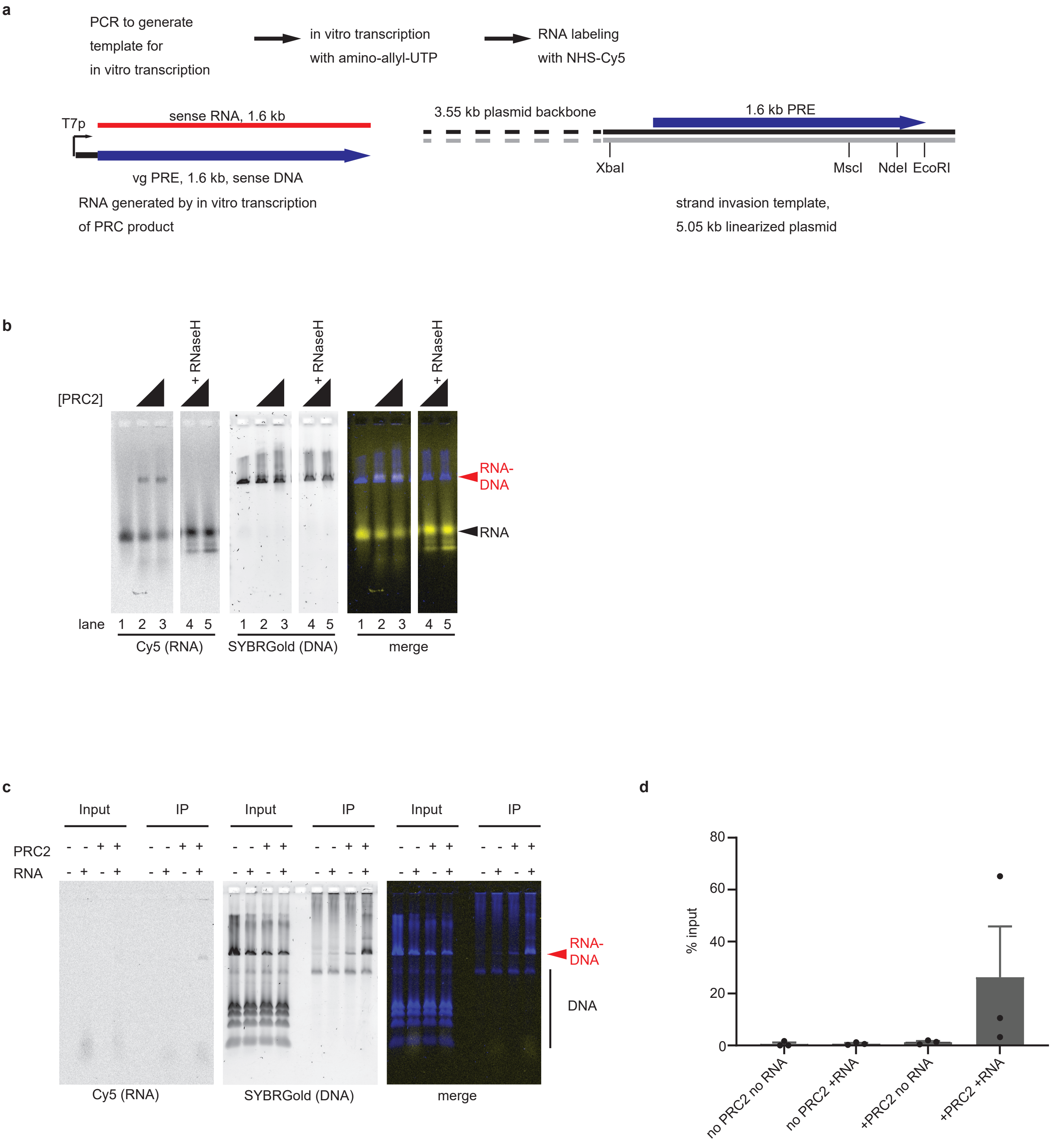
a
PCR to generate template for in vitro transcription
in vitro transcription with amino-allyl-UTP
RNA labeling with NHS-Cy5
T7p
sense RNA, 1.6 kb
vg PRE, 1.6 kb, sense DNA
RNA generated by in vitro transcription of PRC product
3.55 kb plasmid backbone
1.6 kb PRE
XbaI
MscI
NdeI
EcoRI
strand invasion template, 5.05 kb linearized plasmid
b
[PRC2]
+ RNaseH
RNA-DNA
RNA
lane
1 2 3 4 5
Cy5 (RNA)
SYBRGold (DNA)
merge
c
Input
IP
PRC2
RNA
RNA-DNA
DNA
Cy5 (RNA)
SYBRGold (DNA)
merge
d
% input
80
60
40
20
0
no PRC2 no RNA
no PRC2 +RNA
+PRC2 no RNA
+PRC2 +RNA

Supplementary Fig. 6 PRC2 induces the formation of RNA-DNA hybrids that are sensitive to RNaseH and recognized by S9.6. a. Scheme for generating fluorescently labelled RNAs. DNA corresponding to the PRE sequence is amplified with primers, one of which contains the T7 promoter sequence for in vitro transcription. RNAs are produced by in vitro transcription in the presence of amino-allyl UTP, purified, and labelled with NHS-Cy5. These RNAs correspond precisely to the PRE sequence in the DNA template, and are used for strand exchange with linearized, PRE-containing plasmid DNA. Dashed lines indicate plasmid backbone sequence, which is not drawn to scale. Note that the radio-labelled RNAs used in Fig. 3 were generated by in vitro transcription of the circular plasmid template, so they all share ~200 bases of plasmid sequence between the T7 promoter and start of the PRE. b. Representative gels of PRC2 strand exchange activity with Cy5-labelled RNA. PRC2 strand invasion products (lanes 2, 3) are sensitive to RNaseH (lanes 4, 5). [PRC2]=50 and 200 nM, [DNA]=0.3 nM, and [RNA]=0.18 nM. c. Representative gel of DNA incubated with PRC2 and Cy5-labelled RNA after purification and immunoprecipitation with S9.6 antibody. Only the sample containing PRC2, RNA and DNA forms RNA-DNA hybrids that are immunoprecipitated with S9.6. [PRC2]=100 nM, [DNA]=0.3 nM and [RNA]=0.18 nM. d. Quantification of three experiments; error bars are S.E.M. Source data are provided in Source Data file.

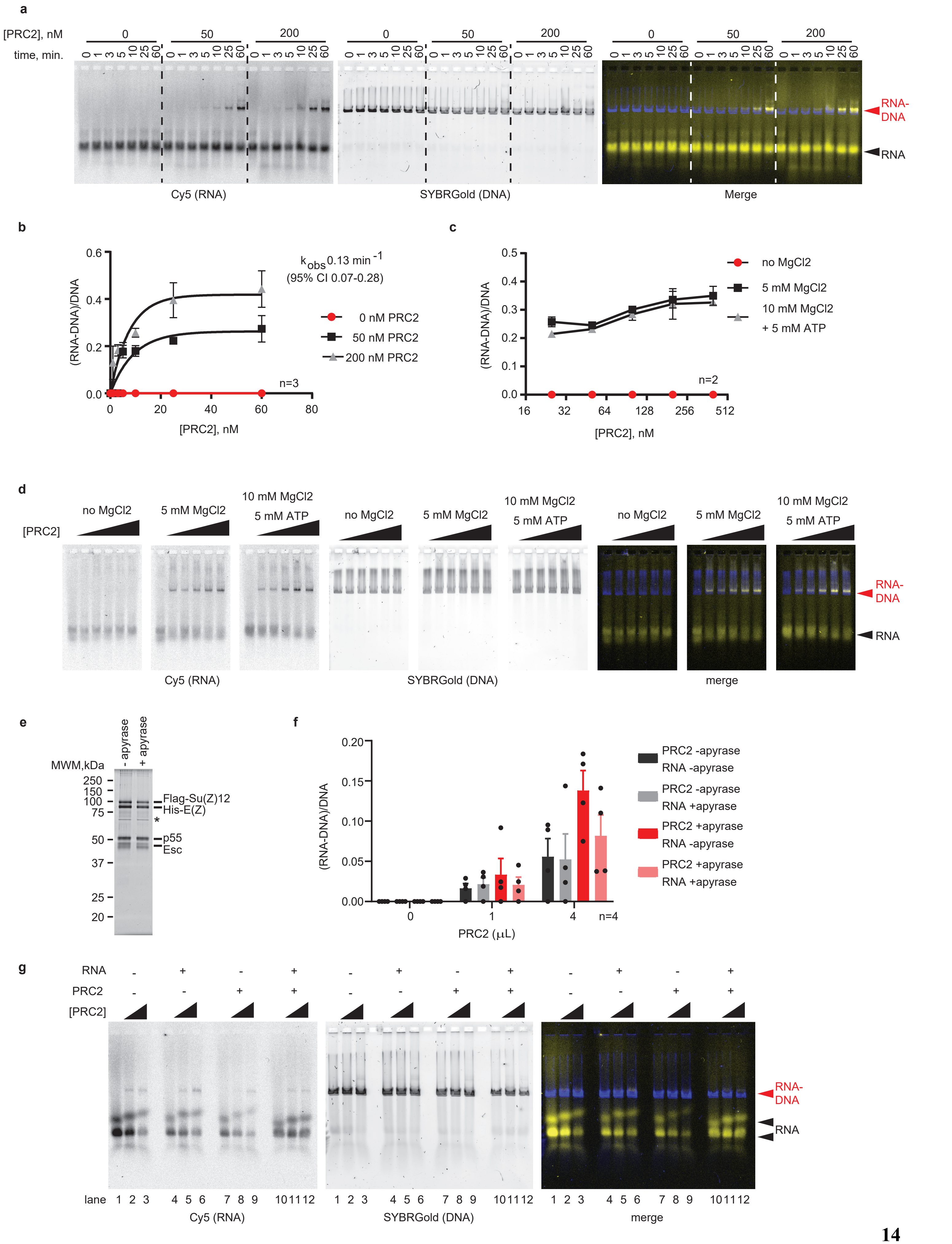
a
[PRC2], nM
time, min.
0 50 200
0 1 3 5 10 25 60
Cy5 (RNA)
SYBRGold (DNA)
Merge
RNA-DNA
RNA
b
(RNA-DNA)/DNA
k_{obs} 0.13 min^{-1}
(95% CI 0.07-0.28)
0 nM PRC2
50 nM PRC2
200 nM PRC2
n=3
[PRC2], nM
c
no MgCl2
5 mM MgCl2
10 mM MgCl2
+ 5 mM ATP
n=2
[PRC2], nM
d
no MgCl2
5 mM MgCl2
10 mM MgCl2
5 mM ATP
[PRC2]
Cy5 (RNA)
SYBRGold (DNA)
merge
e
- apyrase
+ apyrase
MWM,kDa
250 150 100 75 50 37 25 20
Flag-Su(Z)12
His-E(Z)
*
p55
Esc
f
(RNA-DNA)/DNA
PRC2 -apyrase RNA -apyrase
PRC2 -apyrase RNA +apyrase
PRC2 +apyrase RNA -apyrase
PRC2 +apyrase RNA +apyrase
PRC2 (μL)
n=4
g
RNA
PRC2
[PRC2]
lane 1 2 3 4 5 6 7 8 9 10 11 12
Cy5 (RNA)
SYBRGold (DNA)
merge
RNA-DNA
RNA

Supplementary Fig. 7 Time course of PRC2-mediated strand invasion and lack of requirement for nucleotides. a,b. Representative gels (a) and quantification (b) of time course experiment for strand exchange activity. c,d. Quantification (c) and representative gels (d) of RNA-DNA strand exchange activity demonstrating that strand exchange requires $MgCl_2$ but not ATP. PRC2 titration is 25-400 nM. e. SYPRO Ruby-stained SDS-PAGE gel of PRC2 treated with apyrase. *HSC-70 contaminant. f, g. Quantification (f) and representative gels (g) of strand exchange assays carried out with PRC2 and/or RNA that was pre-treated with apyrase to remove any contaminating ATP. Lanes 1-3 PRC2 and RNA not incubated with apyrase, lanes 4-6 RNA pre-treated with apyrase, lanes 7-9 PRC2 pre-treated with apyrase, lanes 10-12 PRC2 and RNA pre-treated with apyrase. Error bars in all graphs are S.E.M. Source data are provided in Source Data file.

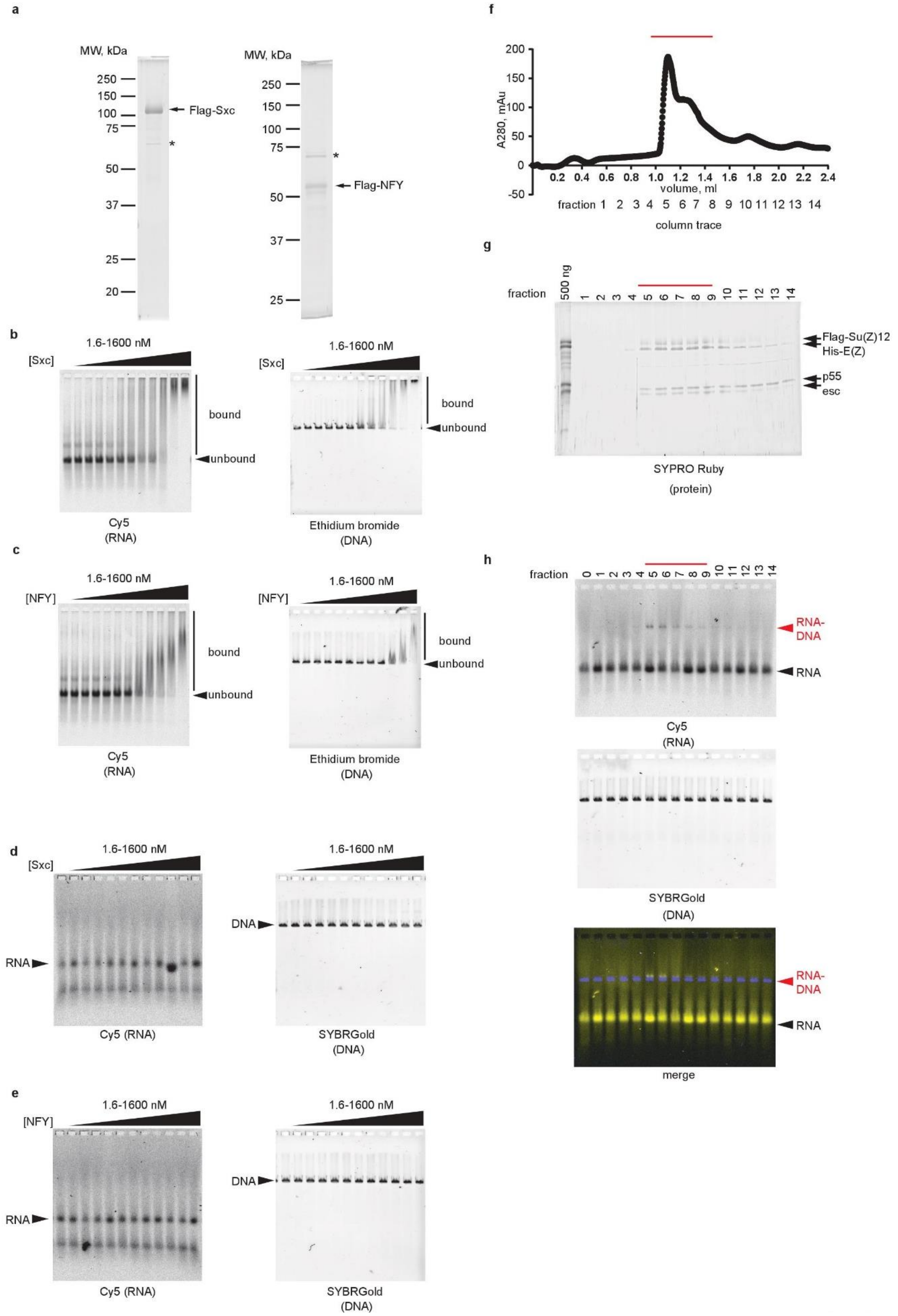
a
MW, kDa
250
150
100
75
50
37
25
20
Flag-Sxc
*
MW, kDa
250
150
100
75
50
37
25
Flag-NFY
*
b
1.6-1600 nM
[Sxc]
bound
unbound
Cy5
(RNA)
1.6-1600 nM
[Sxc]
bound
unbound
Ethidium bromide
(DNA)
c
1.6-1600 nM
[NFY]
bound
unbound
Cy5
(RNA)
1.6-1600 nM
[NFY]
bound
unbound
Ethidium bromide
(DNA)
d
1.6-1600 nM
[Sxc]
RNA
Cy5 (RNA)
1.6-1600 nM
DNA
SYBRGold
(DNA)
e
1.6-1600 nM
[NFY]
RNA
Cy5 (RNA)
1.6-1600 nM
DNA
SYBRGold
(DNA)
f
A280, mAu
200
150
100
50
0
-50
0.2 0.4 0.6 0.8 1.0 1.2 1.4 1.6 1.8 2.0 2.2 2.4
volume, ml
fraction 1 2 3 4 5 6 7 8 9 10 11 12 13 14
column trace
g
fraction
500 ng
1 2 3 4 5 6 7 8 9 10 11 12 13 14
Flag-Su(Z)12
His-E(Z)
p55
esc
SYPRO Ruby
(protein)
h
fraction
0 1 2 3 4 5 6 7 8 9 10 11 12 13 14
RNA-DNA
RNA
Cy5
(RNA)
SYBRGold
(DNA)
RNA-DNA
RNA
merge

Supplementary Fig. 8 Other RNA and DNA binding proteins do not have strand exchange activity and activity co-fractionates with PRC2. a. SYPRO Ruby-stained SDS-PAGE gels of Flag-Sxc and Flag-NFY. *=HSC70 contaminant. b,c. EMSA demonstrating that both Sxc (b) and NFY (c) bind RNA (left) and linear DNA (right), the substrates for strand exchange. d, e. Neither Sxc (d) nor NFY (e) have strand exchange activity at concentrations where they can bind to both substrates. f. 45 µg of PRC2-Flag-Su(Z)12) were fractionated on a Superdex 200 PC3.2/300 column. g. SDS-PAGE (10%) of fractions from size exclusion column. 7 µl of each fraction were loaded on the gel, which was stained with SYPRO Ruby. h. RNA strand exchange assay of the size column fractions. 4 µl of each fraction was used in this assay with 0.3 nM DNA and 0.18 nM RNA. Similar co-fractionation of activity with PRC2 was observed with two additional preparations of PRC2 using glycerol gradient sedimentation, and a second PRC2 preparation using size exclusion chromatography.

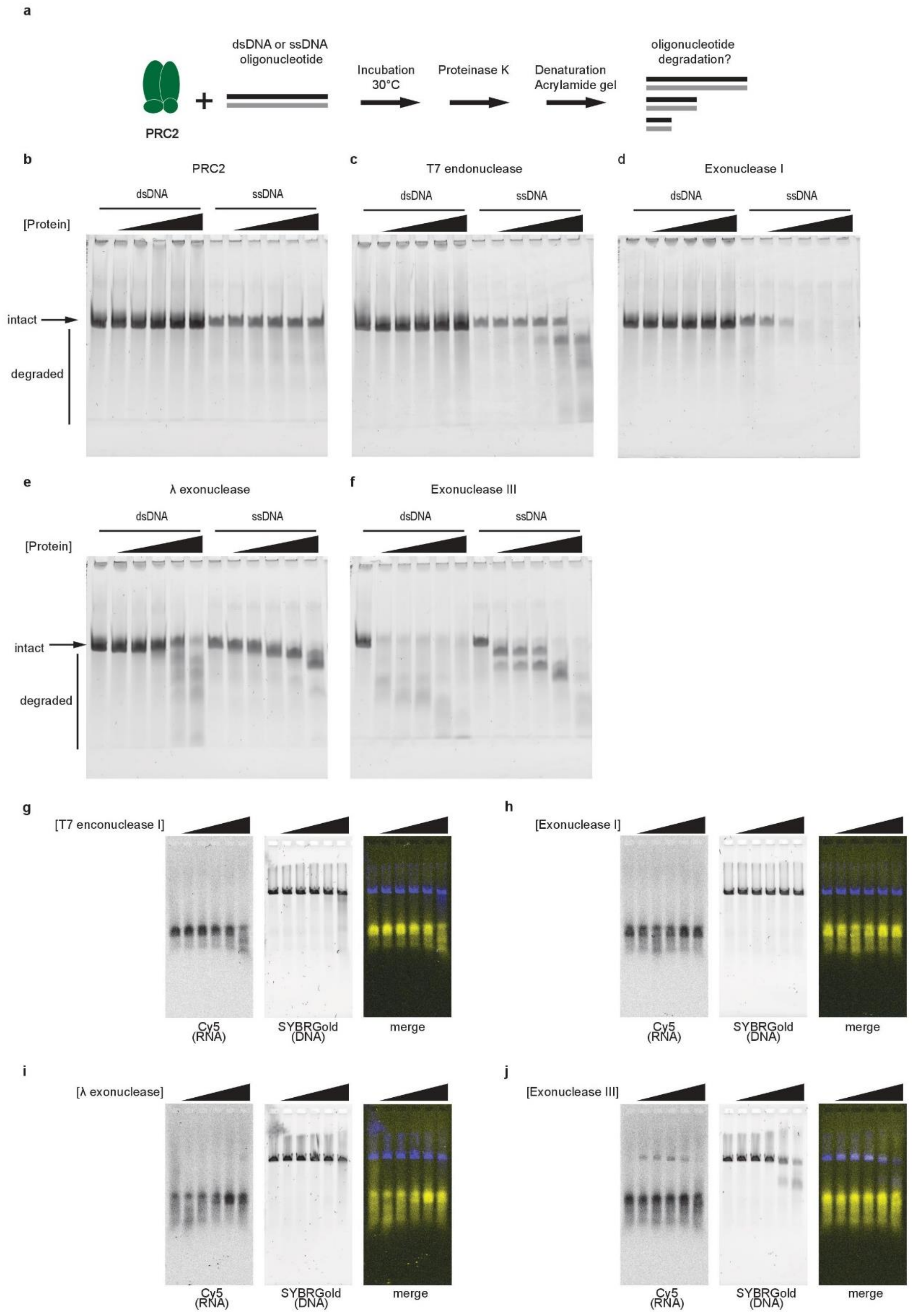
a
dsDNA or ssDNA
oligonucleotide
Incubation
30°C
Proteinase K
Denaturation
Acrylamide gel
oligonucleotide
degradation?
PRC2
b
PRC2
c
T7 endonuclease
d
Exonuclease I
dsDNA
ssDNA
[Protein]
intact
degraded
e
λ exonuclease
f
Exonuclease III
g
[T7 enconuclease I]
Cy5
(RNA)
SYBRGold
(DNA)
merge
h
[Exonuclease I]
i
[λ exonuclease]
j
[Exonuclease III]

Supplementary Fig. 9 PRC2 preparations do not contain detectable nuclease activity. a. Scheme of the experiment testing whether PRC2 preparations contain contaminant nuclease activity. b-f. Representative gels of phosphorylated dsDNA or ssDNA oligonucleotides after incubation with PRC2 (b, n=3), T7 endonuclease I (c, n=3), exonuclease I (d, n=3), λ exonuclease (e, n=3) or exonuclease III (f, n=3) incubated using strand exchange reaction conditions. g-j. Effects of different nucleases on oligonucleotide substrates under the same conditions used for PRC2-induced RNA-DNA strand exchange. Representative gels of DNA and Cy5-labelled RNA incubated with T7 endonuclease I (g), exonuclease I (h), λ exonuclease (i) or exonuclease III (j) (n=2). In all panels the titrations are [PRC2]: 25-400 nM, [T7 endonuclease I]: 0.00004-4 units, [exonuclease I]: 0.00008-8 units, [λ exonuclease]: 0.00002-2 units, [exonuclease III]: 0.0004-40 units.

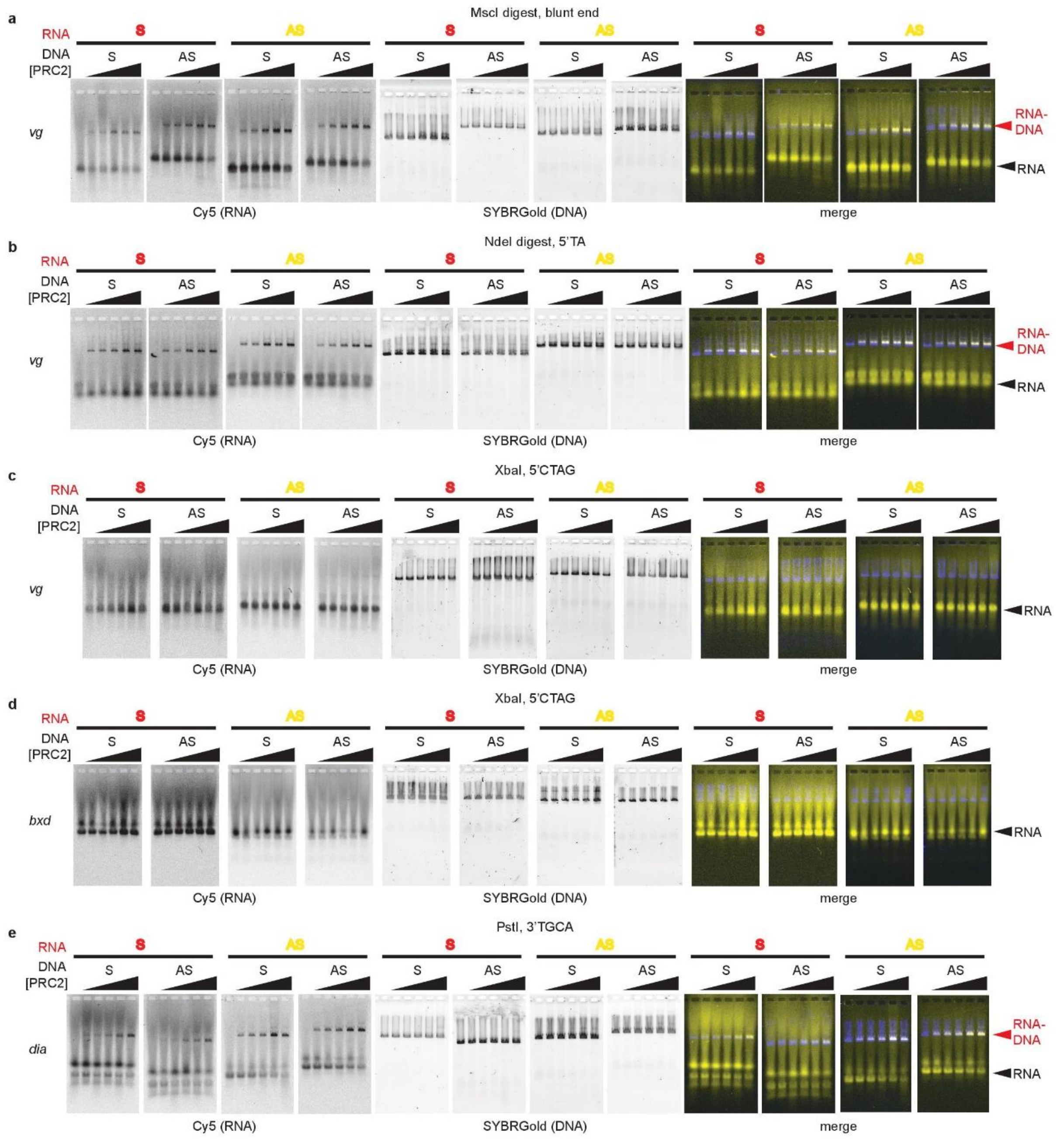
a
MscI digest, blunt end
RNA
S
AS
DNA
S
AS
[PRC2]
vg
RNA-DNA
RNA
Cy5 (RNA)
SYBRGold (DNA)
merge
b
NdeI digest, 5'TA
c
XbaI, 5'CTAG
d
XbaI, 5'CTAG
bxd
e
PstI, 3'TGCA
dia

Supplementary Fig. 10 PRC2 RNA strand exchange activity on different PRE templates with different DNA ends. a-e. Representative gels of RNA strand exchange with different DNA templates and RNAs. PRC2 can induce the formation of RNA:DNA hybrids when provide blunt (a), 5' overhang (b) or 3' overhang (e) DNA end. RNA strand exchange on the *vg* (c), or *bxd* (d) PRE template linearized with XbaI, which is 231 base pairs upstream of where the RNA initiates. Note that both *bxd* RNAs are active in strand exchange with other DNA templates (Fig. 5)**.** In all panels, [PRC2] is 25-400 nM. Experiments were repeated 3 times.

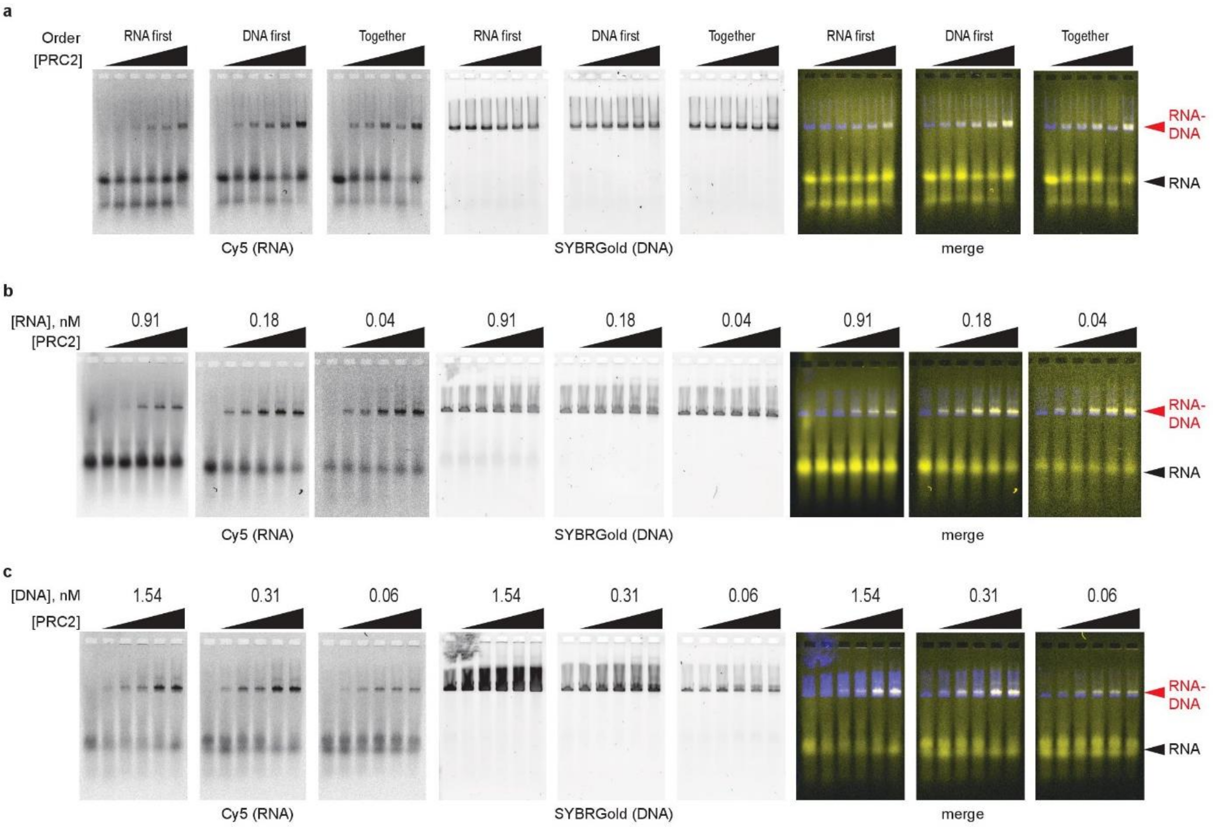
a
Order
[PRC2]
RNA first
DNA first
Together
RNA first
DNA first
Together
RNA first
DNA first
Together
RNA-DNA
RNA
Cy5 (RNA)
SYBRGold (DNA)
merge
b
[RNA], nM
[PRC2]
0.91
0.18
0.04
0.91
0.18
0.04
0.91
0.18
0.04
RNA-DNA
RNA
Cy5 (RNA)
SYBRGold (DNA)
merge
c
[DNA], nM
[PRC2]
1.54
0.31
0.06
1.54
0.31
0.06
1.54
0.31
0.06
RNA-DNA
RNA
Cy5 (RNA)
SYBRGold (DNA)
merge

Supplementary Fig. 11 Effect of order of addition and substrate concentration on PRC2-mediated strand exchange. a. Gels from Fig. 6a (order of addition of DNA and RNA). b. Gels from Fig. 6b (RNA titration). c. Gels from Fig. 6c (DNA titration).

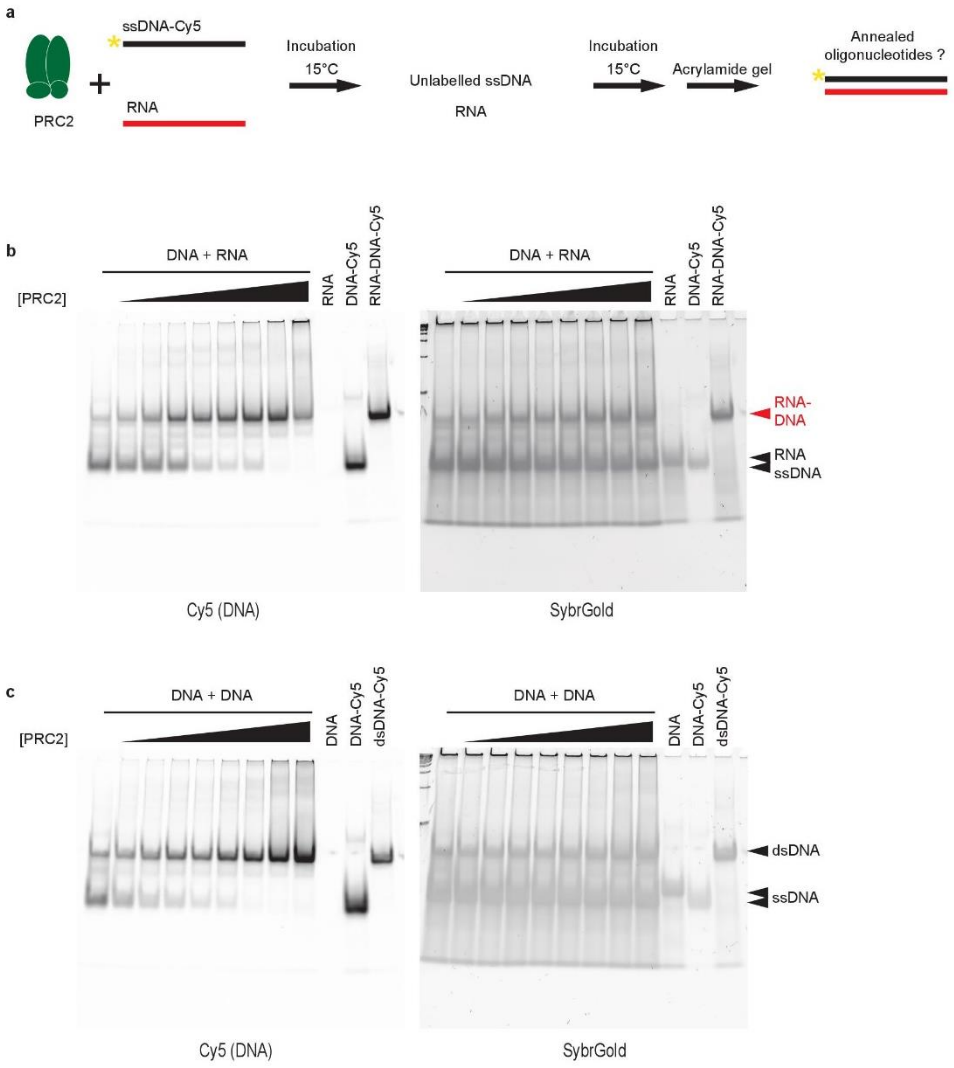
a
ssDNA-Cy5
RNA
PRC2
Incubation
15°C
Unlabelled ssDNA
RNA
Incubation
15°C
Acrylamide gel
Annealed
oligonucleotides ?
b
DNA + RNA
[PRC2]
RNA
DNA-Cy5
RNA-DNA-Cy5
RNA-
DNA
RNA
ssDNA
Cy5 (DNA)
SybrGold
c
DNA + DNA
DNA
DNA-Cy5
dsDNA-Cy5
dsDNA
ssDNA
Cy5 (DNA)
SybrGold

Supplementary Fig. 12 PRC2 can anneal ssDNA and RNA to form RNA:DNA hybrids. a. Scheme for testing annealing activity of PRC2. b. PRC2 can annealed ssDNA and RNA leading to the formation of RNA:DNA hybrid (n=2). c. PRC2 can annealed two ssDNA to form dsDNA (n=2).

**Sequences of primers used for DRIP-qPCR**	
**Primer name**	**Sequence**
Antp F	CAGCATCGCTCTCACTCTCT
Antp R	CAGAAGCAGCGGCTGCAAAA
bxd F	CCATAAGAAATGCCACTTTGC
bxd R	CTCTCACTCTCTCACTGTGAT
vg F	GGCCTCACTTAAGCGCGAAA
vg R	GAGGCGGATCAGCGTTCGAT
dek F	GCGATGAGCCAGAAGATGAG
dek R	CTTGGACTCATCAGTGGCAT
Non1 F	GCGTCCTGTACTTCATGGAT
Non1 R	CTTGTTGATGGCCAGGATGA
tRNA lys F	GCCAAGCTCATTTTCTACGATCT
tRNA lys R	GTCCGACAACGCCGATGATA
dia F	CGGCTCATCTGGAGGAGAAA
dia R	GCGGTGGCATGGGTATGTTT
chic F	AGAGATGGAGAGAGAGTGGC
chic R	GAACGGGTGACTCACGGCTT
dac1 F	GAGAGAGCGGGAGGATTTGA
dac1 R	TTTCTGTGTGCAACCCCGAA
dac2 F	GATACCGAGGGAGCGAAAGA
dac2 R	TGCAAATTTCAGCACTGCGC
sdr F	ACAGCTGATGTCGCTCACAT
sdr R	CGCTGAATGATCACCAGGTGA
en F	GGGTGGTGCACTTTGCAGTT
en R	CGCTGCGATGGGCCAAATAA
Untranscribed F	TCAAGCCGAACCCTCTAAAAT
Untranscribed R	AACGCCAACAAACAGAAAATG
**Sequences of oligonucleotides used to test for nuclease activity and in annealing assay**	
**Primer name**	**Sequence**
bxd PHO core U, DNA or RNA	GAGGCCATCTCAGTCGCACTTAAAACGGCCATTACGAACGA CAGTTATGGCGACGGAGCTGCAGA
bxd PHO core L	TCTGCAGCTCCGTCGCCATAACTGTCGTTCGTAATGGCCGTT TTAAGTGCGACTGAGATGGCCTC
**Sequences of oligonucleotides assembled into R-loop and used in filter binding assay and EMSA**	
**Primer name**	**Sequence**
vgcorePRE RNA F Cy5	TGTGAGTGTGTGCGAGCTGCCAAGTGTGTGTGAGTGTGTT
vgcorePRE DNA R Cy5 or Cy3	GAGCACTTACATGAGCTTGCGTTTGTCACA AACACACTCACACACACTTGGCAGCTCGCACACACTCACA GAAATGGCCGTTAAAATTCTTGACAACCCG

vgcorePRE DNA F	CGGGTTGTCAAGAATTTTAACGGCCATTTC TGTGAGTGTGTGCGAGCTGCCAAGTGTGTGTGAGTGTGTT TGTGACAAACGCAAGCTCATGTAAGTGCTC
vgcorePRE DNA random F	CGGGTTGTCAAGAATTTTAACGGCCATTTC ATAGCTCACCATCATTTGTCGACAATTACTGTCTACTAAT TGTGACAAACGCAAGCTCATGTAAGTGCTC
**Name**	**Primers**
DNA bubble	vgcorePRE DNA R Cy3 / vgcorePRE DNA random F
R-loop	vgcorePRE RNA F Cy5 / vgcorePRE DNA R Cy3 / vgcorePRE DNA random F
dsDNA	vgcorePRE DNA R Cy3 / vgcorePRE DNA F
ssDNA	vgcorePRE DNA F

Supplementary Table 1 Sequences of primers and oligonucleotides used

Chromosome	start	end
chr2L	5410	6069
chr2L	368280	369380
chr2L	377320	378520
chr2L	585040	585960
chr2L	594180	595780
chr2L	597440	598540
chr2L	817270	817869
chr2L	1954140	1955540
chr2L	1966960	1967960
chr2L	1969980	1971360
chr2L	1972160	1973500
chr2L	2093620	2094269
chr2L	2449340	2450460
chr2L	2454260	2455360
chr2L	2754860	2755780
chr2L	2828680	2829309
chr2L	2900140	2900779
chr2L	3538120	3540780
chr2L	3545000	3545900
chr2L	3571080	3572160
chr2L	3580520	3581560
chr2L	3606060	3607460
chr2L	3657160	3658580
chr2L	3661720	3663140
chr2L	3824900	3826140
chr2L	3834800	3836020
chr2L	3836080	3837280
chr2L	4809740	4812340
chr2L	5403080	5404460
chr2L	5421240	5422360
chr2L	5460640	5462100
chr2L	5923280	5924400
chr2L	5924760	5925940
chr2L	5979930	5980659
chr2L	6019440	6020540
chr2L	6535680	6536860
chr2L	6545070	6545849
chr2L	6821160	6822400
chr2L	6833200	6834480
chr2L	7302040	7303060
chr2L	7305740	7307600
chr2L	8862880	8863509
chr2L	9581260	9582460
chr2L	9607720	9608720

chr2L	10076810	10077499
chr2L	11358000	11359180
chr2L	11445160	11446940
chr2L	11447060	11448480
chr2L	12003280	12005020
chr2L	12005240	12008020
chr2L	12009620	12011919
chr2L	12085360	12086500
chr2L	12586520	12587640
chr2L	12617520	12619080
chr2L	12655420	12656520
chr2L	12677920	12679040
chr2L	12986910	12987539
chr2L	13098100	13098639
chr2L	14409920	14410980
chr2L	14489740	14491300
chr2L	15110920	15112100
chr2L	15333085	15334320
chr2L	15339280	15340320
chr2L	16422117	16423740
chr2L	16485320	16488220
chr2L	16532500	16533380
chr2L	16982270	16983139
chr2L	18735160	18736320
chr2L	18736960	18737920
chr2L	18782360	18784040
chr2L	18795500	18797180
chr2L	18880740	18882260
chr2L	18935740	18936860
chr2L	19108200	19109200
chr2L	19259290	19259909
chr2L	19768700	19770440
chr2L	20764200	20765119
chr2L	20769940	20771240
chr2L	20775303	20778340
chr2L	20782720	20783840
chr2L	21789160	21790180
chr2L	21827700	21828960
chr2L	21851100	21852260
chr2L	21866100	21867100
chr2L	21868380	21869860
chr2L	21898800	21899840
chr2L	22024020	22025220
chr2R	1613860	1615120
chr2R	2993620	2994500
chr2R	3230830	3231549
chr2R	3305560	3307320

chr2R	3316300	3317440
chr2R	3533450	3533959
chr2R	3905880	3907160
chr2R	3908280	3909640
chr2R	3911520	3912620
chr2R	3917780	3919180
chr2R	3938360	3939960
chr2R	4159660	4160720
chr2R	5081960	5083160
chr2R	5490190	5491169
chr2R	5866000	5867320
chr2R	5875400	5876600
chr2R	6690880	6691539
chr2R	7043840	7044419
chr2R	7078830	7079359
chr2R	7356580	7358045
chr2R	7362342	7363954
chr2R	7364840	7365900
chr2R	7408390	7409159
chr2R	7414940	7417800
chr2R	7429180	7430220
chr2R	7439140	7440160
chr2R	7446560	7447600
chr2R	7448200	7449100
chr2R	7450060	7451100
chr2R	7452680	7453860
chr2R	7469160	7470080
chr2R	8420990	8421599
chr2R	8705580	8706359
chr2R	8765500	8766209
chr2R	8771360	8772600
chr2R	8792050	8793631
chr2R	8842420	8848140
chr2R	8859860	8860419
chr2R	8867680	8869100
chr2R	8871820	8873900
chr2R	8877900	8879860
chr2R	8881080	8881779
chr2R	8883280	8885220
chr2R	8924771	8933108
chr2R	9209830	9210669
chr2R	10095080	10096160
chr2R	10343260	10344320
chr2R	10345020	10346440
chr2R	10364760	10365740
chr2R	10473680	10474479
chr2R	10686120	10687660

chr2R	10845181	10848201
chr2R	11216300	11217460
chr2R	11722160	11723920
chr2R	11793210	11793899
chr2R	12794540	12795099
chr2R	12817340	12818079
chr2R	13644260	13645240
chr2R	13709300	13711440
chr2R	13824250	13825109
chr2R	14405190	14405879
chr2R	14481120	14482059
chr2R	14793480	14794009
chr2R	14807810	14808569
chr2R	14853393	14856461
chr2R	15150680	15151820
chr2R	15162500	15163640
chr2R	15953920	15954439
chr2R	16220960	16221609
chr2R	16442560	16443189
chr2R	16443800	16444740
chr2R	16451040	16451589
chr2R	16738130	16738749
chr2R	16790560	16791800
chr2R	16800680	16803040
chr2R	16803340	16804740
chr2R	16847980	16849520
chr2R	16861560	16862379
chr2R	16950710	16951279
chr2R	17266540	17267520
chr2R	17565210	17565799
chr2R	18129020	18131920
chr2R	18157740	18158840
chr2R	18506210	18506809
chr2R	18747300	18748400
chr2R	18754800	18755960
chr2R	18930180	18931200
chr2R	18933200	18934320
chr2R	18981500	18982460
chr2R	19467880	19468880
chr2R	19508500	19509660
chr2R	19519340	19520480
chr2R	19534200	19535280
chr2R	19805730	19806469
chr2R	19954770	19955469
chr2R	20353010	20353629
chr2R	20699220	20700200
chr2R	20700800	20702840

chr2R	20739120	20740240
chr2R	20939540	20940700
chr2R	20948720	20949900
chr2R	20987240	20988860
chr2R	21002560	21003840
chr2R	21101880	21103420
chr3L	361380	362119
chr3L	370880	371960
chr3L	376800	378860
chr3L	432240	433320
chr3L	699220	700120
chr3L	1110470	1111329
chr3L	1176900	1178880
chr3L	1349370	1349969
chr3L	1919800	1921680
chr3L	1962500	1963109
chr3L	2904980	2905639
chr3L	3624480	3625540
chr3L	3625620	3626740
chr3L	3845360	3846720
chr3L	3855160	3856420
chr3L	3876520	3877400
chr3L	3878420	3879460
chr3L	5874080	5875680
chr3L	6090680	6091459
chr3L	6782380	6783740
chr3L	6788690	6789399
chr3L	6877860	6878589
chr3L	6896720	6898000
chr3L	6981580	6983200
chr3L	7198990	7199489
chr3L	7230560	7231169
chr3L	7827940	7828749
chr3L	7921670	7922189
chr3L	7951440	7952380
chr3L	8997420	8998720
chr3L	9011920	9013120
chr3L	9033620	9034960
chr3L	9930040	9930759
chr3L	10218740	10219980
chr3L	10282000	10283300
chr3L	10713710	10714469
chr3L	11001540	11004360
chr3L	11447200	11447849
chr3L	11920000	11921180
chr3L	12321440	12322640
chr3L	12426480	12428300

chr3L	12460680	12461920
chr3L	12572840	12574200
chr3L	12583620	12584600
chr3L	12601940	12603240
chr3L	12620380	12621420
chr3L	12645860	12647020
chr3L	12683420	12684560
chr3L	12685340	12687180
chr3L	13393840	13394840
chr3L	14124120	14125560
chr3L	14125580	14127261
chr3L	14142920	14143980
chr3L	14170200	14171720
chr3L	14545630	14546239
chr3L	14571580	14572820
chr3L	14592220	14594420
chr3L	14761660	14762539
chr3L	15036620	15037780
chr3L	17094070	17094869
chr3L	17100230	17100989
chr3L	17123050	17123569
chr3L	17499420	17500219
chr3L	17650650	17651389
chr3L	18392620	18394080
chr3L	19499760	19500609
chr3L	20629150	20629879
chr3L	21469180	21470620
chr3L	21472320	21473540
chr3L	22249520	22250419
chr3R	166560	167580
chr3R	171980	173140
chr3R	175620	176800
chr3R	659060	660060
chr3R	660400	661740
chr3R	677600	679000
chr3R	1108260	1108759
chr3R	2356480	2357099
chr3R	2503560	2505040
chr3R	2508520	2509620
chr3R	2551420	2552340
chr3R	2561620	2575018
chr3R	2616320	2618040
chr3R	2631280	2632240
chr3R	2636020	2637040
chr3R	2656551	2661848
chr3R	2673820	2675140
chr3R	2675220	2676380

chr3R	2706860	2708280
chr3R	2711640	2713052
chr3R	2718380	2721410
chr3R	2728500	2729980
chr3R	2738120	2739760
chr3R	2747020	2747980
chr3R	2755920	2756840
chr3R	2763040	2764240
chr3R	2825820	2827340
chr3R	2862660	2863509
chr3R	2961360	2962300
chr3R	3414450	3415189
chr3R	3788100	3789860
chr3R	4006660	4008220
chr3R	4016160	4017360
chr3R	4103160	4104180
chr3R	4159080	4161400
chr3R	4246540	4247309
chr3R	4528660	4529189
chr3R	4898960	4899559
chr3R	5058020	5058839
chr3R	5124740	5125379
chr3R	6001750	6002339
chr3R	6193600	6194760
chr3R	6361180	6362180
chr3R	6439600	6441160
chr3R	6442020	6443520
chr3R	6447500	6448600
chr3R	6490340	6491480
chr3R	7128280	7129360
chr3R	7137620	7138520
chr3R	7178800	7179960
chr3R	7296770	7297279
chr3R	8069800	8070840
chr3R	8104300	8105420
chr3R	8106100	8107280
chr3R	8837630	8838559
chr3R	8882680	8883820
chr3R	8889060	8891840
chr3R	9638040	9639180
chr3R	9700240	9701500
chr3R	9702440	9703620
chr3R	9720980	9722160
chr3R	9726800	9728040
chr3R	9748620	9749980
chr3R	9755640	9756720
chr3R	9973680	9974249

chr3R	10547800	10548559
chr3R	10604850	10605369
chr3R	10854680	10855399
chr3R	11222160	11222899
chr3R	11818420	11819620
chr3R	11834460	11836380
chr3R	11839020	11840020
chr3R	11851270	11852049
chr3R	11863320	11864500
chr3R	11947050	11947549
chr3R	12210900	12212080
chr3R	12228800	12230220
chr3R	12239140	12240240
chr3R	12248660	12249920
chr3R	12487100	12488160
chr3R	12503400	12504400
chr3R	12527147	12529718
chr3R	12543920	12544880
chr3R	12545340	12546520
chr3R	12589020	12590917
chr3R	12619900	12620900
chr3R	12636230	12638320
chr3R	12641040	12642120
chr3R	12644800	12645920
chr3R	12655380	12656500
chr3R	12662960	12664020
chr3R	12672560	12673700
chr3R	12674100	12675060
chr3R	12677440	12678400
chr3R	12682960	12684200
chr3R	12694584	12695860
chr3R	12707720	12709000
chr3R	12711800	12712960
chr3R	12722831	12726610
chr3R	12738860	12739980
chr3R	12744380	12746860
chr3R	12748920	12750040
chr3R	12759500	12760700
chr3R	12763480	12764520
chr3R	12765960	12766980
chr3R	12773860	12775080
chr3R	12778380	12779300
chr3R	12783240	12784780
chr3R	12785560	12786780
chr3R	12789000	12789920
chr3R	12794760	12796620
chr3R	12800100	12801020

chr3R	12808380	12809500
chr3R	13382860	13385060
chr3R	13392160	13393160
chr3R	13665160	13665709
chr3R	13769340	13770280
chr3R	14061260	14062340
chr3R	14196820	14197920
chr3R	14524740	14526129
chr3R	14538050	14538809
chr3R	15662120	15663120
chr3R	16672200	16673220
chr3R	17204060	17205260
chr3R	17259420	17260740
chr3R	17271600	17272940
chr3R	17324640	17326000
chr3R	17382440	17384280
chr3R	17386860	17388060
chr3R	18751520	18752039
chr3R	18957657	18960119
chr3R	18967140	18968380
chr3R	18968720	18969920
chr3R	19018720	19019820
chr3R	19022740	19023640
chr3R	19026380	19027620
chr3R	19032620	19033680
chr3R	19036960	19038360
chr3R	19118340	19119520
chr3R	19120680	19121780
chr3R	19122580	19123540
chr3R	20274110	20274859
chr3R	20412020	20412759
chr3R	20563690	20564199
chr3R	20906070	20906699
chr3R	21514670	21515599
chr3R	21846160	21847860
chr3R	21924480	21925480
chr3R	22376610	22377489
chr3R	22415140	22415789
chr3R	22612790	22613499
chr3R	23343810	23344579
chr3R	23543670	23544339
chr3R	23989520	23990089
chr3R	24069750	24070279
chr3R	24074310	24074839
chr3R	24133610	24134229
chr3R	24372480	24373700
chr3R	24410580	24411940

chr3R	25378140	25379260
chr3R	25381260	25382600
chr3R	25397270	25398620
chr3R	25515680	25518740
chr3R	25598120	25599000
chr3R	25855500	25856580
chr3R	26098320	26100420
chr3R	26441780	26443020
chr3R	26543020	26543759
chr3R	26562800	26563559
chr3R	26589380	26590640
chr3R	26590920	26591920
chr3R	26592360	26593560
chr3R	26598160	26599240
chr3R	26606380	26608340
chr3R	26677180	26678440
chr3R	26734440	26735480
chr3R	26737460	26738960
chr3R	26887620	26888840
chr3R	27363230	27363909
chr3R	27897320	27898599
chrX	482620	483720
chrX	484100	485380
chrX	1465980	1466709
chrX	1751910	1752599
chrX	1832030	1832699
chrX	1846700	1847580
chrX	2016880	2021172
chrX	2030422	2033330
chrX	2036680	2037760
chrX	2322722	2324624
chrX	2327298	2330636
chrX	2764990	2765779
chrX	3153980	3154819
chrX	4271780	4272289
chrX	4304880	4306240
chrX	4307790	4308509
chrX	4317420	4319900
chrX	4320260	4321640
chrX	4401760	4403100
chrX	4420540	4421520
chrX	4448460	4449440
chrX	4483500	4484900
chrX	4490140	4491660
chrX	4510340	4512020
chrX	4556370	4556929
chrX	4881030	4881839

chrX	5104440	5105159
chrX	5157100	5157819
chrX	5454060	5455400
chrX	5457980	5458900
chrX	5473000	5474360
chrX	5487500	5488700
chrX	5894400	5895860
chrX	5906800	5908140
chrX	6353870	6354789
chrX	6379120	6379979
chrX	7004520	7005620
chrX	7119740	7120700
chrX	7449730	7450249
chrX	7520260	7521740
chrX	8041710	8042469
chrX	8171430	8172269
chrX	8324250	8324869
chrX	8348840	8349720
chrX	8530040	8531600
chrX	8534320	8535580
chrX	8544240	8546060
chrX	8570280	8571360
chrX	8651440	8652560
chrX	8699380	8702020
chrX	8769530	8770209
chrX	9430870	9431389
chrX	9586340	9588680
chrX	9622680	9624080
chrX	9691870	9692389
chrX	9767360	9768219
chrX	10500130	10500759
chrX	10862540	10863149
chrX	10948390	10949239
chrX	11097230	11098069
chrX	11560680	11561359
chrX	11974910	11975689
chrX	12316580	12317209
chrX	12449570	12450279
chrX	12483460	12484069
chrX	12509420	12510560
chrX	12671680	12672439
chrX	12755880	12756409
chrX	13820470	13821059
chrX	13871990	13872759
chrX	13888440	13889880
chrX	14337960	14338739
chrX	14804700	14805199

chrX	15249980	15251200
chrX	16041160	16042580
chrX	16042640	16043820
chrX	16044160	16045380
chrX	16050930	16051639
chrX	16110200	16112300
chrX	17037000	17037809
chrX	17191780	17192529
chrX	17207020	17209000
chrX	17216120	17216839
chrX	17239520	17240740
chrX	17290740	17291900
chrX	17601930	17602789
chrX	17658620	17659640
chrX	17661660	17663100
chrX	17682700	17684960
chrX	17696060	17697080
chrX	18077030	18077889
chrX	18202900	18204220
chrX	18757770	18758559
chrX	19289800	19290599
chrX	19429300	19430280
chrX	19439560	19441460
chrX	20560060	20562260
chrX	20564560	20565920
chrX	20747910	20748549
chrX	20825720	20826239

Supplementary Table 2 List of PRES used in this work

**Data set**	**Source**
RNA-seq embryos	SRA009364
RNA-seq S2 cells	GSE72830
All_EST	UCSC (D. melanogaster ESTs - D. melanogaster ESTs Including Unspliced), dm3
PREs	Predicted PREs [53]
	Conserved PcG binding sites [54]
	vg PRE [16]
	list of PREs [55]
	dac PRE [46]
ChIP-seq, 4-12 hour embryos (Ph, PC, H3K27me3, DSP1) (merge of 2 replicates) (*18*)	GSE60428 (used to compare to both 2-6H and 10-14H R-loop data)
ChIP-seq, H3K27Ac, H3K27me3, RNA pol II 0-4H, 4-8H, 8-12H, 12-16H embryos	GSE16013
ChIP-seq, S2 cells (E(Z), dRING, GAF)	GSE101554
ChIP-seq, S2 cells H3K27Ac, H3K27me3	modENCODE_326; GSE41440
ChIP-chip, S2 cells, RNA pol II	modENCODE_3295

Supplementary Table 3 Data sets used for comparison with DRIP-seq data
