# Supplementary material for: RNA-DNA strand exchange by the Drosophila Polycomb complex PRC2
Source: Nat Commun. 2020 Apr 14;11:1781. doi: 10.1038/s41467-020-15609-x (PMC7156742; doi:10.1038/s41467-020-15609-x)
Supplement: Supplementary file 3 — Reporting Summary [file 41467_2020_15609_MOESM3_ESM.pdf]

natureresearch

Corresponding author(s): Nicole J Francis

Last updated by author(s): Feb 21, 2020

Reporting Summary

Nature Research wishes to improve the reproducibility of the work that we publish. This form provides structure for consistency and transparency in reporting. For further information on Nature Research policies, see Authors & Referees and the Editorial Policy Checklist.

Statistics

For all statistical analyses, confirm that the following items are present in the figure legend, table legend, main text, or Methods section.

n/a	Confirmed	
☐	☒	The exact sample size (n) for each experimental group/condition, given as a discrete number and unit of measurement
☒	☐	A statement on whether measurements were taken from distinct samples or whether the same sample was measured repeatedly
☐	☒	The statistical test(s) used AND whether they are one- or two-sided<br>*Only common tests should be described solely by name; describe more complex techniques in the Methods section.*
☒	☐	A description of all covariates tested
☐	☒	A description of any assumptions or corrections, such as tests of normality and adjustment for multiple comparisons
☐	☒	A full description of the statistical parameters including central tendency (e.g. means) or other basic estimates (e.g. regression coefficient) AND variation (e.g. standard deviation) or associated estimates of uncertainty (e.g. confidence intervals)
☐	☒	For null hypothesis testing, the test statistic (e.g. F, t, r) with confidence intervals, effect sizes, degrees of freedom and P value noted<br>*Give P values as exact values whenever suitable.*
☒	☐	For Bayesian analysis, information on the choice of priors and Markov chain Monte Carlo settings
☒	☐	For hierarchical and complex designs, identification of the appropriate level for tests and full reporting of outcomes
☒	☐	Estimates of effect sizes (e.g. Cohen's d, Pearson's r), indicating how they were calculated

Our web collection on statistics for biologists contains articles on many of the points above.

Software and code

Policy information about availability of computer code

Data collection	No software was used in data collection.
Data analysis	Trimmomatic (ChiP-seq pipeline), Bourgey et al., 2019<br>Samtools v 1.4.1, Li et al., 2009<br>BEDtools, Quinlan and Hall, 2010<br>DeepTools v 2.5.3, Ramirez, Dundar, Diehl, Gruning, Manke, 2014<br>Picard v2.17.3, http://broadinstitute.github.io/picard<br>Bowtie2 v 2/2.3.1, Langmead and Salzberg, 2012<br>MACS2 v 2.1.1, Zhang et al., 2009<br>Galaxy, Afgan et al., 2018, https://usegalaxy.org/<br>Pavis, Huang et al., 2013, https://manticore.niehs.nih.gov/pavis2/<br>RegioneR, Gel B, Diez-et al. (2016). doi: 10.1093/bioinformatics/btv562.<br>GraphPad Prism 8<br>ImageQuantTL, GE Healthcare<br>UCSC LiftOver<br>SRA tooolkit v2.9.6 (http://ncbi.github.io/sra-tools/, SRA Toolkit Development Team<br>BEDOPs v2.4.34, Neph et al., 2012

For manuscripts utilizing custom algorithms or software that are central to the research but not yet described in published literature, software must be made available to editors/reviewers. We strongly encourage code deposition in a community repository (e.g. GitHub). See the Nature Research guidelines for submitting code & software for further information.

Data

Policy information about availability of data

All manuscripts must include a data availability statement. This statement should provide the following information, where applicable:

- Accession codes, unique identifiers, or web links for publicly available datasets
- A list of figures that have associated raw data
- A description of any restrictions on data availability

Sequence (DRIP-seq) data that support the findings of this study have been deposited in NCBI GEO with the accession code GSE127329. Public data sets used for this analysis are listed in Supplementary Table 3. The source data underlying Figures 2c, 2d, 3g, 4f, 6b, 6d, and 6f and Extended Data Figures 1b, 6d, 7b, 7c and 7c are provided as a Source Data file. Other data that support the findings of this study are available from the corresponding author upon reasonable request.

Field-specific reporting

Please select the one below that is the best fit for your research. If you are not sure, read the appropriate sections before making your selection.

☒ Life sciences ☐ Behavioural & social sciences ☐ Ecological, evolutionary & environmental sciences

For a reference copy of the document with all sections, see nature.com/documents/nr-reporting-summary-flat.pdf

Life sciences study design

All studies must disclose on these points even when the disclosure is negative.

Sample size	Biochemical experiments were typically conducted at least three times (independently) with two different preparations of protein. In cases where a smaller sample size is used, it is because a similar but non-identical experiment produced the same result. For genome-wide analysis, two replicates were conducted. The replicates were well correlated with each other and only peaks identified in both data sets were used.
Data exclusions	For binding data, points that were clearly technical aberrations were excluded. These are indicated in the Source Data files.
Replication	Experiments were conducted multiple times (as described above). For biochemical experiments we have typically also done multiple versions of each type of experiment (for example different templates).
Randomization	not relevant to the study.
Blinding	not relevant to the study.

Reporting for specific materials, systems and methods

We require information from authors about some types of materials, experimental systems and methods used in many studies. Here, indicate whether each material, system or method listed is relevant to your study. If you are not sure if a list item applies to your research, read the appropriate section before selecting a response.

Materials & experimental systems

n/a	Involved in the study	
☐	☒	Antibodies
☐	☒	Eukaryotic cell lines
☒	☐	Palaeontology
☐	☒	Animals and other organisms
☒	☐	Human research participants
☒	☐	Clinical data

Methods

n/a	Involved in the study	
☐	☒	ChIP-seq
☒	☐	Flow cytometry
☒	☐	MRI-based neuroimaging

Antibodies

Antibodies used	S9.6 antibody was obtained from Kerafast (lot # 072616) and from Dr. Michael Wilson.
Validation	The S9.6 antibody has been extensively validated in the literature, but has also been controversial because it is able to recognize dsRNA (Hartano et al, J. Mol. Biol., 2018), and may not recognize all R-loops equally, independent of sequence (Konig et al., PLoS One, 2017). To address binding to RNA, we extensively digest our purified (R-loop containing) genomic DNA with RNAseA and RNase III. To confirm that the signal in our DRIP experiments is derived from R-loops, we run RNaseH treated samples in parallel with all S9.6 DRIP experiments, and only consider signals that are sensitive to RNaseH (and thus contain RNA-DNA hybrids) in our analysis. A control pull down with a transcribed plasmid that is digested into an R-loop containing and R-loop free region is also run in each experiment to confirm that the antibody highly enriches R-loop containing DNA. We cannot rule out the possibility

that our data sets are biased towards R-loops formed from sequences that are preferentially recognized by S9.6 (thus we may underestimate the number of R-loops in the genome). This does not substantially alter the conclusions of our paper.

Eukaryotic cell lines

Policy information about cell lines

Cell line source(s)	Drosophila S2 Cells, ThermoFisher, SKU# R690-07
Authentication	https://www.thermofisher.com/order/catalog/product/R69007#/R69007<br>Quality and performance testing<br>Each lot of Gibco® S2 cells is tested for cell growth and viability post-recovery from cryopreservation. In addition, the Master Seed Bank has been tested for contamination of bacteria, yeast, mycoplasma and virus and has been characterized by isozyme and karyotype analysis.
Mycoplasma contamination	mycoplasma contamination was not tested
Commonly misidentified lines<br>(See ICLAC register)	N/R

Animals and other organisms

Policy information about studies involving animals; ARRIVE guidelines recommended for reporting animal research

Laboratory animals	Drosophila melanogaster, Oregon R
Wild animals	This study did not involve wild animals
Field-collected samples	This study did not involve field-collected samples
Ethics oversight	No ethical approval or guidance was required for this study.

Note that full information on the approval of the study protocol must also be provided in the manuscript.

ChIP-seq

Data deposition

☒ Confirm that both raw and final processed data have been deposited in a public database such as GEO.

☒ Confirm that you have deposited or provided access to graph files (e.g. BED files) for the called peaks.

Data access links
May remain private before publication.

NCBI GEO with the accession code GSE127329

Files in database submission

PROCESSED DATA FILES
file name file type
2_6a_DRIP.bw bigwig
2_6a_DRIP_F.bw bigwig
2_6a_DRIP_R.bw bigwig
2_6b_DRIP.bw bigwig
2_6b_DRIP_F.bw bigwig
2_6b_DRIP_R.bw bigwig
2_6a_DRIP_RNH.bw bigwig
2_6a_DRIP_RNH_F.bw bigwig
2_6a_DRIP_RNH_R.bw bigwig
2_6b_DRIP_RNH.bw bigwig
2_6b_DRIP_RNH_F.bw bigwig
2_6b_DRIP_RNH_R.bw bigwig
2_6a_IN.bw bigwig
2_6a_IN_F.bw bigwig
2_6a_IN_R.bw bigwig
2_6b_IN.bw bigwig
2_6b_IN_F.bw bigwig
2_6b_IN_R.bw bigwig
10_14a_DRIP.bw bigwig
10_14a_DRIP_F.bw bigwig
10_14a_DRIP_R.bw bigwig
10_14b_DRIP.bw bigwig
10_14b_DRIP_F.bw bigwig
10_14b_DRIP_R.bw bigwig
10_14a_DRIP_RNH.bw bigwig
10_14a_DRIP_RNH_F.bw bigwig

10_14a_DRIP_RNH_R.bw bigwig
10_14b_DRIP_RNH.bw bigwig
10_14b_DRIP_RNH_F.bw bigwig
10_14b_DRIP_RNH_R.bw bigwig
10_14a_IN.bw bigwig
10_14a_IN_F.bw bigwig
10_14a_IN_R.bw bigwig
10_14b_IN.bw bigwig
10_14b_IN_F.bw bigwig
10_14b_IN_R.bw bigwig
S2a_DRIP.bw bigwig
S2a_DRIP_F.bw bigwig
S2a_DRIP_R.bw bigwig
S2b_DRIP.bw bigwig
S2b_DRIP_F.bw bigwig
S2b_DRIP_R.bw bigwig
S2a_DRIP_RNH.bw bigwig
S2a_DRIP_RNH_F.bw bigwig
S2a_DRIP_RNH_R.bw bigwig
S2b_DRIP_RNH.bw bigwig
S2b_DRIP_RNH_F.bw bigwig
S2b_DRIP_RNH_R.bw bigwig
S2a_IN.bw bigwig
S2a_IN_F.bw bigwig
S2a_IN_R.bw bigwig
S2b_IN.bw bigwig
S2b_IN_F.bw bigwig
S2b_IN_R.bw bigwig
2-6H_F.bed bed
2-6H_R.bed bed
2-6H_unstr.bed bed
10-14H_F.bed bed
10-14H_R.bed bed
10-14H_unstr.bed bed
S2_F.bed bed
S2_R.bed bed
S2_unstr.bed bed
RAW FILES
file name file type
2-6Ha_IP_R1.fastq.gz FastQ
2-6Ha_IP_R2.fastq.gz FastQ
2-6Hb_IP_R1.fastq.gz FastQ
2-6Hb_IP_R2.fastq.gz FastQ
2-6Ha_RNH_IP_R1.fastq.gz FastQ
2-6Ha_RNH_IP_R2.fastq.gz FastQ
2-6Hb_RNH_IP_R1.fastq.gz FastQ
2-6Hb_RNH_IP_R2.fastq.gz FastQ
2-6Ha_Input_R1.fastq.gz FastQ
2-6Ha_Input_R2.fastq.gz FastQ
2-6Hb_Input_R1.fastq.gz FastQ
2-6Hb_Input_R2.fastq.gz FastQ
10-14Ha_IP_R1.fastq.gz FastQ
10-14Ha_IP_R2.fastq.gz FastQ
10-14Hb_IP_R1.fastq.gz FastQ
10-14Hb_IP_R2.fastq.gz FastQ
10-14Ha_RNH_IP_R1.fastq.gz FastQ
10-14Ha_RNH_IP_R2.fastq.gz FastQ
10-14Hb_RNH_IP_R1.fastq.gz FastQ
10-14Hb_RNH_IP_R2.fastq.gz FastQ
10-14Ha_Input_R1.fastq.gz FastQ
10-14Ha_Input_R2.fastq.gz FastQ
10-14Hb_Input_R1.fastq.gz FastQ
10-14Hb_Input_R2.fastq.gz FastQ
S2a_IP_R1.fastq.gz FastQ
S2a_IP_R2.fastq.gz FastQ
S2b_IP_R1.fastq.gz FastQ
S2b_IP_R2.fastq.gz FastQ
S2a_RNH_IP_R1.fastq.gz FastQ
S2a_RNH_IP_R2.fastq.gz FastQ
S2b_RNH_IP_R1.fastq.gz FastQ
S2b_RNH_IP_R2.fastq.gz FastQ
S2a_Input_R1.fastq.gz FastQ
S2a_Input_R2.fastq.gz FastQ
S2b_Input_R1.fastq.gz FastQ

S2b_Input_R2.fastq.gz FastQ

Genome browser session
(e.g. UCSC)

N/R

Methodology

Replicates

Two biological replicates were carried out for each DRIP-seq data set. The correlation between the replicates was examined using multiBigwigSummary on Galaxy (bin size: 1000 bp) followed by plotCorrelation using the Pearson correlation method. Correlations for replicates were: 2-6H 0.97, 10-14H 0.87, S2 0.99.

Sequencing depth

Paired end reads were used for all DRIP-seq.
Sample,# reads,% aligned reads,% single alignment,% multiply aligned,fraction duplicates removed
S2a_IN,70115200,93.07,65.51,27.01,0.12
S2a_DRIP,78536947,90.73,49.18,41.25,0.22
S2a_RNH,76251872,,,,0.32
S2b_IN,49491097,94.02,64.67,29,0.08
S2b_DRIP,50416335,92.29,53.39,36.7,0.11
S2b_RNH,44494974,45.29,21.06,24.13,0.38
2-6Ha_IN,59134272,94.51,65.91,28.17,0.084
2-6Ha_DRIP,71304601,92.13,40.39,51.49,0.31
2-6Ha_RNH,67314471,72.13,43.54,28.49,0.356
2-6Hb_IN,98964609,90.96,63.26,26.99,0.24
2-6Hb_DRIP,81784635,90.43,28.88,61.21,0.48
2-6Hb_RNH,57601406,30.05,13.97,15.88,0.499
10-14Ha_IN,53768480,93.55,65.6,27.45,0.09
10-14Ha_DRIP,70498991,89.76,44.01,45.46,0.31
10-14Ha_RNH,10803247,38.69,23.11,15.52,0.23
10-14Hb_IN,74331700,92.27,65.05,26.63,0.17
10-14Hb_DRIP,140216626,87.02,40.27,46.46,0.38
10-14Hb_RNH,41859858,43.6,22.54,20.88,0.45

Antibodies

S9.6 antibody was obtained from Kerafast (lot # 072616) and from Dr. Michael Wilson.

Peak calling parameters

Peaks were called for DRIP versus input and DRIP versus RNaseH treated using MACS250 (v. 2.1.1) (-f BAMPE --bw 250 -g dm --mfold 10 30 -q 0.01). For strand specific peaks, strand specific files were used (e.g. F-strand DRIP, F-strand input, F-strand RNaseH). Peaks present in both DRIP vs. input and DRIP vs. RNaseH were retained (BEDTools intersect) 51 for each duplicate. Finally, BEDTools (intersect) was used to retain only peaks present in both duplicates, which were used for further analysis.

Data quality

FDR was set to 0.01 for all peak calling.

Software

Software used is indicated in the methods.
Trimmomatic (ChiP-seq pipeline), Bourgey et al., 2019
Samtools v 1.4.1, Li et al., 2009
BEDtools, Quinlan and Hall, 2010
DeepTools v 2.5.3, Ramirez, Dundar, Diehl, Gruning, Manke, 2014
Picard v2.17.3, http://broadinstitute.github.io/picard
Bowtie2 v 2/2.3.1, Langmead and Salzberg, 2012
MACS2 v 2.1.1, Zhang et al., 2009
Galaxy, Afgan et al., 2018, https://usegalaxy.org/
Pavis, Huang et al., 2013, https://manticore.niehs.nih.gov/pavis2/
RegioneR, Gel B, Diez-et al. (2016). doi: 10.1093/bioinformatics/btv562.
GraphPad Prism 8
ImageQuantTL, GE Healthcare
UCSC LiftOver
SRA tooolkit v2.9.6 (http://ncbi.github.io/sra-tools/, SRA Toolkit Development Team
BEDOPs v2.4.34, Neph et al., 2012
